# Pollination crisis Down‐Under: Has Australasia dodged the bullet?

**DOI:** 10.1002/ece3.10639

**Published:** 2023-10-30

**Authors:** Graham H. Pyke, Kit S. Prendergast, Zong‐Xin Ren

**Affiliations:** ^1^ CAS Key Laboratory for Plant Diversity and Biogeography of East Asia Kunming Institute of Botany, Chinese Academy of Sciences Kunming China; ^2^ School of Natural Sciences Macquarie University Ryde New South Wales Australia; ^3^ School of Biological Sciences & Biotechnology Murdoch University Perth Western Australia Australia

**Keywords:** agricultural intensification, crop pollination, food security, pesticide use, pollination services, pollinator decline, threatened species, urbanization

## Abstract

Since mid‐1990s, concerns have increased about a human‐induced “pollination crisis.” Threats have been identified to animals that act as plant pollinators, plants pollinated by these animals, and consequently human well‐being. Threatening processes include loss of natural habitat, climate change, pesticide use, pathogen spread, and introduced species. However, concern has mostly been during last 10–15 years and from Europe and North America, with Australasia, known as Down‐Under, receiving little attention. So perhaps Australasia has “dodged the bullet”? We systematically reviewed the published literature relating to the “pollination crisis” via Web of Science, focusing on issues amenable to this approach. Across these issues, we found a steep increase in publications over the last few decades and a major geographic bias towards Europe and North America, with relatively little attention in Australasia. While publications from Australasia are underrepresented, factors responsible elsewhere for causing the “pollination crisis” commonly occur in Australasia, so this lack of coverage probably reflects a lack of awareness rather than the absence of a problem. In other words, Australasia has not “dodged the bullet” and should take immediate action to address and mitigate its own “pollination crisis.” Sensible steps would include increased taxonomic work on suspected plant pollinators, protection for pollinator populations threatened with extinction, establishing long‐term monitoring of plant–pollinator relationships, incorporating pollination into sustainable agriculture, restricting the use of various pesticides, adopting an Integrated Pest and Pollinator Management approach, and developing partnerships with First Nations peoples for research, conservation and management of plants and their pollinators. Appropriate Government policy, funding and regulation could help.

## Graphical abstract

The pollination crisis has relatively little attention in Australasia or Down Under, in comparison with Europe and North America, suggesting that this region may have escaped the problem or dodged the bullet. However, factors underlying the pollination crisis commonly occur in Australasia, and so the lack of concern there reflects lack of awareness and is not justified. Consequently, Australasia should immediately address and mitigate its own pollination crisis, facilitated through policy, regulation, and funding.

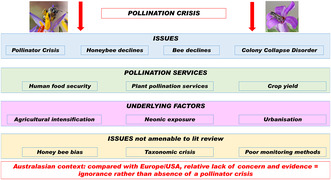

## 1 INTRODUCTION

Since the mid‐1990s, scientists have raised concerns that the world is facing a “pollination crisis” involving threats to animals that act as plant pollinators, to the plants that these animals pollinate, and to human well‐being (Bartomeus et al., 2019; Buchmann & Nabhan, 1996b; Holden, 2006). Concern has been expressed, for example, in regard to declines in pollinators and the pollination services that they provide (Rhodes, 2018; Vieli et al., 2021), and to consequent impacts on plant reproduction, both for wild and crop species (Fantinato & Buffa, 2019; Lopez‐Cubillos et al., 2021).

There is, furthermore, general agreement that this “pollination crisis” is serious and needs to be resolved, especially as it results from human activities that influence the nature and extent of natural habitat for plants and animals; cause their declines and extinctions; and lead to climate change, over/misuse of pesticides, spread of pathogens, and spread of introduced species (managed or established in the wild) (Dicks et al., 2021). Typically, for example, as human numbers and use of natural resources have increased, with associated increased urbanization, agriculture and use of fossil fuel, we have seen extensive changes to natural vegetation, the climate, species distributions and abundances, and environmental pollution, which combine to threaten humanity, and the natural world upon which it depends, with a potentially “ghastly future” (Bradshaw et al., 2021). The pollination crisis is one manifestation of this general dilemma and warrants attention.

Recent articles and other publications further indicate that concern regarding the pollination crisis is currently surging, but predominantly in Europe and North America, and much less elsewhere. There are, for example, rapidly increasing numbers of published articles, largely arising out of Europe and North America, that express such concern in the context of pollinator decline (Millard et al., 2020), effects of pesticides on honeybees (Abati et al., 2021), effects of pesticides on solitary bees (Lehmann & Camp, 2021), effects of urbanization on native bees (Prendergast, Dixon, et al., 2022b), and pollination management in protected cropping environments (Egan et al., 2020; Kendall et al., 2021; Lundin et al., 2021).

In this article, we review the literature regarding the pollination crisis, distinguishing between the various issues involved, and paying particular attention to temporal trends and possible geographic biases. In addition, as this crisis seems to have been largely neglected in Australasia, we focus on this region, known colloquially and in the literature (e.g., Verloop et al., 2020) as “Down‐Under,” which includes Australia, New Zealand (NZ), New Guinea, and other nearby islands (i.e., Fiji, New Caledonia, Norfolk Island, Solomon Islands, and Vanuatu). Thus, we undertook a targeted review of the literature regarding the pollination crisis, based on specific search terms in academic databases (see below), and extended this review with additional relevant articles when appropriate. Thus, our review was not intended to be comprehensive, but was detailed, focused, clear and objective, and could be replicated.

## 2 METHODS

We review the “pollination crisis” based primarily on literature searches carried out using the Web of Science (WoS from now on), supplemented with information from other sources (i.e., FAOSTAT website, Discover Life website) as well as literature review datasets from previous similar searches carried out by other authors (Prendergast, Dixon, et al., 2022b; Siviter, Richman, et al., 2021). We supplemented these literature searches with additional reading of articles as they arose, for example, by following citation trails. For our WoS searches, we adopted the “All Databases” option and chose combinations of search “Topics” that addressed particular issues, were reasonably simple, and generated moderate and manageable numbers of articles, with a low proportion that were unrelated to any aspect of the “pollination crisis” and therefore considered irrelevant (see below and Appendix S1 for examples of such topics). For our searches the end search date was always December 31, 2021; two searches carried out by other authors had earlier end dates (see Appendix S1: supplementary text). As we found that the results of our WoS searches depended on both the chosen search option (i.e., Core vs. All Databases) and the institutional base of the person carrying out the search, we report all such details in Appendix S1.

For each article generated by our searches, we excluded duplicate articles and then focused on article relevance to the issue of interest, publication year, and associated country. We determined article relevance (i.e., simply relevant, in the context of pollination crisis, or not) on the basis of any abstract or summary and, if necessary but rarely, the main text. Consequently, determining article relevance rarely required downloading and viewing it. Those articles that were considered irrelevant were excluded. Year of publication was always included in the search output for each article. We considered the country associated with a particular article to be either where the study was carried out if it involved data collection, the first‐mentioned study location if data were collected in more than one location, and the first‐listed location for the first‐listed author if the article was a review or opinion, or otherwise did not involve data collection. We thus determined the associated country on the basis of the list of authors and their addresses as well as the main text, especially any methods section. Consequently, determining associated country for an article generally required downloading and viewing it. Downloading and viewing articles required significantly more time per article than simply reading the titles and abstracts, so we aimed to limit the extent to which we had to do this.

For articles that met our criteria for inclusion, we tallied publication years for all articles generated by each search, but tallied associated countries and regions (i.e., continental areas) for just the latest several years. For determining countries and regions we aimed to include enough recent years so that we ended up with at least 100 relevant articles to consider, but when the total number of relevant articles was fewer than 100, we assessed country and region for all of them. We also assumed that the inclusion of some irrelevant articles would not significantly affect the pattern of year‐to‐year variation, but might affect tabulation of associated countries and regions. If an article was not duplicated but appeared twice in our search output, because it was first published online and early and subsequently published with a new date, we used only the most recent publication.

We present details regarding WoS and other searches in the Appendix S1 – Text. Such additional information also includes, for each issue of interest, a spreadsheet containing the citation, publication year, and associated region for articles considered relevant and fully processed (see Appendix S2 – Spreadsheet, Worksheets #01 to #13). For the Australasian region, we present details separately for Australia, NZ, and the rest of Australasia.

We present some additional details regarding our methods as they arise below.

## 3 REVIEW

### 3.1 Concern regarding the “pollination crisis”

Not surprisingly, the “pollination crisis” has been the subject of much concern, but attention to this problem has mostly been very recent and has arisen primarily in North America and Europe, with little attention in Australasia. For example, using the WoS to search the literature for articles referring to either “pollination crisis” or “pollinator crisis,” we found that 63 (i.e.,72%) of 87 such articles were based on work carried out in Europe or North America, with just one article for NZ, none for Australia, and none for the rest of Australasia (Table 1; Appendix S1 – Text: Issue #01 – Table S01‐1). We also found that the annual publication rate for these articles has increased greatly, especially since about 2005 (Table 1; Appendix S1 – Text: Issue #01 – Table S01‐2).

**TABLE 1:** Summaries for geographic region and year period for articles from searches relating to the “pollination crisis” and component crises.

Issue (issue #)	Source	Region (% articles)										Year period (articles/year; # years = 5, unless otherwise indicated)								
		N1	Europe	North America	Asia	South America	Europe, N Amer, Asia	Africa	Aust	N.Z.	Rest of Australasia	N2	1970–1989 (20 years)	1990–1994	1995–1999	2000–2004	2005–2009	2010–2014	2015–2019	2020–2021 (2 years)
Pollination/ pollinator crisis (#01)	WoS	87	41%	29%	16%	10%		2%	0%	1%	0%	87	0	0	0.4	1.6	2.8	4.0	6.4	5.5
Honeybee Declines (#02)	WoS	102	31%	39%	18%	5%		4%	1%	2%	0%	120	0	0.2	0.2	0	1.4	6.8	10.4	10.0
Colony collapse disorder (#03)	WoS	118	31%	35%	18%	12%		3%	3%	0%	0%	383	0	0	0	0	6.2	30.0	30.0	25.5
Declines in bees other than honeybees (#04)	WoS	103	34%	49%	8%	5%		1%	4%	0%	0%	189	0.1	0.2	0.6	0.2	1.8	4.8	17.6	30.0
Threatened bee species (#05)	IUCN Red List	483	83%	11%	0%	5%	1%	0%	0%	0%	0%									
Human food security (#06)	WoS	115	34%	17%	19%	15%		12%	3%	0%	0%	287	0	0	0	0.2	1.2	11.6	25.0	48.5
Declines in pollination services (#07)	WoS	42	38%	29%	14%	7%		2%	5%	5%	0%	42	0	0	0	0.6	0.2	2.4	3.0	5.5
Shortfalls in crop pollination (#08)	WoS	83	33%	18%	16%	22%		7%	4%	0%	1% (Fiji)	42	0.05	0.2	0	0.2	0.4	5.4	6.0	10.5
Combined (#01 to #08, excl #05)	WoS	650	34.0%	31.2%	15.7%	10.9%		4.8%	2.5%	0.8%	0.2%	1191	0.15	0.6	1.4	2.8	14.0	65.0	99.6	135.5

*Note*: All searches, but one, involved the WoS, and were carried out by the authors, unless otherwise indicated. One search was based on the IUCN Red List of threatened species (IUCN, 2022; accessed 15 June 2022). N1 is the number of articles assessed for associated country and region. N2 is the number of articles for which year and year period are tabulated. Australasia is considered to include Australia, Fiji, New Caledonia, New Zealand, Norfolk Island, New Guinea, Solomon Islands, and Vanuatu. Other islands of the South Pacific are included in Oceania, but no articles were associated with this region.

These observations suggest either that the pollination crisis may not apply in Australasia and hence that this region may have “dodged the bullet” in terms of this problem or alternatively that there has been relatively little attention dedicated to the issues around a pollination crisis in Australasia and hence the significance of the problem in Australasia is largely unknown. We consider the evidence for these possibilities below.

### 3.2 Pollination crisis as set of interrelated issues

This pollination crisis is potentially quite complex, involving a set of interrelated issues or “crises,” depending on whether the focus is on the animals that act as pollinators, pollination of the plants involved, or human well‐being. Furthermore, for both plants and animals, populations may be natural (i.e., native and wild), managed (e.g., bees maintained in artificial domiciles, plants grown as cultivated crops), or feral/exotic (i.e., non‐native, established in the wild). There are therefore many potential combinations to consider.

On the other hand, concern regarding the pollination crisis has so far focused mostly on the following issues, which simplifies discussion. One group of issues involves animals that visit and potentially pollinate flowers and focuses on declines in these animals. Though diverse animals, including birds, wasps, flies, beetles, butterflies, moths, bats and possums, can serve as pollinators (Buchmann & Nabhan, 1996a; Ollerton, 2021), we focus our review on bees, as they rely entirely on floral resources for food for their whole life cycle, are major plant pollinators (e.g., Ballantyne et al., 2017; Danforth, 2007), and feature predominantly in discussions relevant to the pollination crisis (see below).

The issues for this group are as follows:

- Declines in honeybees (Issue #02).
- Concern regarding colony collapse disorder, a widely recognised symptom of decline in managed honeybees (e.g., Donkersley et al., 2020; Neov et al., 2021) (Issue #03). Though conceptually related to honeybee decline, this issue has been reasonably distinct in the literature and the two issues generate largely different references.
- Declines in populations and species diversity of wild and native species of other bees (i.e., non‐honeybees) (Issue #04).

We carried out literature searches for these issues using WoS (see Table 1 and Appendix S1 – Text; Issues #02 to #04). We also used information from the IUCN Red List (IUCN, 2022; accessed 15 June 2022) to address the following issue (see Table 1 and Appendix S1 – Text; Issue #05):

- numbers of threatened bee species in different geographic regions.
  A second group of issues considers pollination services provided to plants by these animals. These are
- Human food security in context of pollination (Issue #06).
- Declines in pollination services to plants in general, including species that are wild and native, introduced, and cultivated as crops (Issue #07).
- Shortfalls in crop pollination arising from crop expansion or pollinator decline or both (Issue #08).

We carried out literature searches for these issues using the WoS (see Table 1 and Appendix S1 – Text; Issues #06 to #08).

For each of the above issues, attention has increased markedly since about 2005–2009 and arisen mostly in Europe and North America, with little in Australia and NZ, and none in the rest of Australasia (Table 1; Appendix S1: Issues S01 to S08). Combining the issues covered by WoS searches (i.e., Issues #01 to #08, excl #05), Europe and North America account for 65.2% of articles, while Australia, NZ, and the rest of Australasia account for just 2.5%, 0.8% and 0.2% respectively (Table 1). In other words, Australasia has accounted for just 3.5% of these articles. Similarly combining the same issues, the annual rate of publication of articles has increased greatly, especially since 2005–2009, and is continuing to increase steeply (Table 1).

In addition, according to the IUCN Red List, there are no bee species currently listed as threatened with extinction in Australia, New Zealand, or the rest of Australasia (Table 1; Appendix S1: Issue S05).

These observations reinforce the view that these issues, and other aspects of the pollination crisis, have been under‐represented in Australasia. Is this because Australasia has so far dodged the bullet? We further consider this possibility below.

### 3.3 Underlying factors responsible for the “pollination crisis”

There is general agreement that the above issues of concern in the context of pollination have arisen because of various human‐caused factors including agricultural intensification, urbanization, pesticide‐use, impacts of managed pollinators (typically bees) on wild and native plants and their pollinators, climate change, and habitat alteration and loss. As described below, it was possible to carry out literature searches or use existing searches for most, but not all, of these factors. However, as also described, results were consistent across the factors.

To consider the attention that has been given to evidence of the factors underlying the pollination crisis, we carried out literature searches using WoS for the following two issues (Table 2; Appendix S1 – Text; Issues #09 & #10):

- Impacts of agricultural intensification in context of pollinators and pollination (Issue #09).
- Effects on non‐*Apis* bees from exposure to neonicotinoids (widely used insecticides on crops) (here we updated the WoS survey carried out by Siviter, Johnson, et al., 2021; Siviter, Richman, et al., 2021) (Issue #10).

**TABLE 2:** Summaries for geographic region and year‐period for articles from searches relating to factors responsible for the “pollination crisis”.

Issue (issue #)	Source	Region (% articles)									Year period (articles/year; # years =5, unless otherwise indicated)								
		N1	Europe	North America	Asia	South America	Africa	Aust	N.Z.	Rest of Australasia	N2	1970–1989 (20 years)	1990–1994	1995–1999	2000–2004	2005–2009	2010–2014	2015–2019	2020–2021 (2 years)
Agricultural intensification (#09)	WoS	114	53%	23%	6%	11%	7%	0%	1%	0%	285	0	0	0.2	1.2	5.8	13.2	24.6	30.0
Bees and neonic exposure (#10)	WoS	61	66%	31%	0%	3%	0%	0%	0%	0%	61	0	0	0	0.8	0.2	2.6	5.0	9.0
Urbanization (#11)	WoS	202	37%	42%	5%	10%	1%	6%	0%	0%	202	0	0.4	0.2	1.6	5.6	11.2	21.0	
Combined (#9 & #10)	WoS	175	57.1%	25.7%	8.0%	4.0%	4.6%	0%	0.6%	0%	346	0	0	0.2	2.0	6.0	15.8	29.6	39.0

*Note*: All searches involved the WoS and were carried out by the authors, unless otherwise indicated. N1 is the number of articles assessed for associated country and region. N2 is the number of articles for which year and year‐period are tabulated. Australasia is considered to include Australia, Fiji, New Caledonia, New Zealand, Norfolk Island, New Guinea, Solomon Islands, and Vanuatu. Other islands of the South Pacific are included in Oceania, but there were no articles associated with this region.

We also utilised results from an existing publication for the following issue (Table 2; Appendix S1 – Text; Issue #11):

- Impacts of urbanization in context of pollinators and pollination (search using Google Scholar by Prendergast, Dixon, et al., 2022b) (Issue #11).

However, we were unable to similarly consider some other relevant issues. We were, for example, unable to carry out useful WoS searches, in the context of pollination, for either climate change or habitat alteration/ loss because such searches generated huge numbers of references, with many being irrelevant. We were also unable to assess geographic and year‐to‐year patterns with WoS searches or similar, for articles relevant to impacts of introduced pollinators because native/ wild, feral, and managed bees of the same species (e.g., honeybees, *Apis mellifera*) may occur together, and the natural distribution of the honeybee includes much of Africa, Europe and Asia, but excludes the Americas, Australasia, and Oceania.

There have, however, been many recent articles that consider these factors (e.g., climate change: [Gerard et al., 2020; Soroye et al., 2020]; habitat loss: [Niemuth et al., 2021; Prendergast, Dixon, et al., 2022b]; impacts of introduced pollinators: [Aizen et al., 2020; Debnam et al., 2021; Prendergast, Dixon, et al., 2022a]).

Similar to the identification of the pollination crisis and some of its components and consequent expression of concern, as described above, attention devoted to the underlying factors has been recent and focused primarily in Europe and North America, with little devoted to Australia and NZ, and none to the rest of Australasia based on WoS searches (Table 2; Appendix S1 – Text; Issues #09 to #10). Combining the results of these searches, 82.8% of articles are associated with Europe or North America and only 0.6% with Australasia, and the publication rate of articles has increased markedly since about 2005–2009 (Table 2).

Taken at face value, this scarcity of attention, in Australia and NZ and the rest of Australasia, to the factors generally underlying the pollination crisis suggests that this crisis does not apply to Australasia. However, as argued below, we doubt the validity or wisdom of such an approach.

### 3.4 Other associated crises

Adding to the pollinator crisis, as discussed above, are the following additional crises that exacerbate the problem, especially in Australasia:

- Lack of basic information about plant–pollinator relationships, including which animal species visit flowers of which plant species (as reflected in interest in “pollination networks”) (Issue #12)
- Taxonomic impediment to understanding such relationships, arising from a lack of species descriptions for flower‐visiting animal species, especially for insects, and a lack of taxonomic expertise for describing and identifying such flower‐visiting species (as reflected in mentions of “taxonomic impediment” in the context of pollination) (Issue #13)

WoS searches for these two issues indicate, once again, that interest and concern have surged recently, but mostly in Europe and North America, and very little in Australia, NZ, or the rest of Australasia (Table 3; Appendix S1 – Text; Issues #12 & #13). Combining results of these two searches, 52.9% of articles are associated with Europe and North America and 7.3% with Australasia, and the publication rate of articles has increased from about 2005–2009 (Table 3).

**TABLE 3:** Summaries for geographic region and year period for articles from searches relating to other associated crises.

Issue	Source	Region (% articles)									Year period (articles/year; # years = 5, unless otherwise indicated)								
		N1	Europe	North America	Asia	South America	Africa	Aust	N.Z.	Rest of Australasia	N2	1970–1989 (20 years)	1990–1994	1995–1999	2000–2004	2005–2009	2010–2014	2015–2019	2020–2021 (2 years)
Pollination Networks (Issue #12)	WoS	99	33%	21%	19%	15%	5%	2%	2%	2%	452	0	0.2	0	0.6	7.4	20.8	41.4	50.0
Taxonomic Impediment (Issue #13)	WoS	24	13%	33%	13%	21%	8%	8%	4%	0%	24	0	0	0	0	0.8	0.8	2.0	3.0
Combined (#12 & #13)	WoS	123	29.3%	23.6%	17.9%	16.3%	5.7%	3.3%	2.4%	1.6%	476	0	0.2	0	0.6	8.2	21.6	43.4	53.0

*Note*: All searches involved the WoS and were carried out by the authors, unless otherwise indicated. N1 is the number of articles assessed for associated country and region. N2 is the number of articles for which year and year‐period are tabulated. Australasia is considered to include Australia, Fiji, New Caledonia, New Zealand, Norfolk Island, New Guinea, Solomon Islands, and Vanuatu. Other islands of the South Pacific are included in Oceania, but no articles were associated with this region.

Moreover, though not readily amenable to literature searches, the following related issues compound the pollination crisis:

- Bias towards (European) honeybees in studies on bee declines and their causes

For example, studies of the impacts on pollinators of pesticides and other agricultural chemicals have largely focused on honeybees (Abati et al., 2021; Almeida et al., 2021), with little attention to other bee species (Chan & Raine, 2021; Siviter, Richman, et al., 2021), and to other kinds of potentially pollinating animals such as butterflies (Braak et al., 2018). Similarly, studies of viruses that infect bees have similarly focused on the honeybee (Daughenbaugh et al., 2021). Further such studies of non‐Apis bees and other pollinator species are clearly warranted (Franklin & Raine, 2019; Siviter, Richman, et al., 2021).

- Adoption of the (European) honeybee as the icon for pollinator conservation, in general, and bee conservation, in particular, with associated growth in urban honeybee keeping.
  For example, many people, especially in the general community, have equated the well‐being of managed populations of the (European) honeybee with conservation of bee diversity, amelioration of pollination issues, and the quality of the relationship between humanity and nature, leading to calls to “save the bees,” which has really meant “save the honeybees” (Hall & Martins, 2020; Lorenz, 2021; Marshman & Knezevic, 2021; Sponsler & Bratman, 2021). Furthermore, this perspective of the honeybee has fuelled recent and very large increases in urban honeybee keeping (Sponsler & Bratman, 2021). However, the managed honeybee is under no conservation threat (Halvorson et al., 2021; Osterman et al., 2021), and this focus on it is a distraction from the real issues that form the pollination crisis (Colla, 2022; Colla & MacIvor, 2017; Ollerton et al., 2012).
- Lack of appropriate monitoring of pollinator populations

Determining trends in pollinator abundance, factors that are responsible and potential ways to mitigate any declines require data from standardised monitoring programs, as well as increased availability and utilization of existing monitoring data (Bartomeus & Dicks, 2019; Breeze et al., 2021; O'Connor et al., 2019). Poor choice of monitoring methods, including in Australia, can hamper such monitoring (Portman et al., 2020; Prendergast et al., 2020; Prendergast & Hogendoorn, 2021).

### 3.5 Steps to mitigate the pollination crisis

Recognised ways to mitigate the pollination crisis, apart from dealing with very general problems such as climate change and broadscale degradation of natural vegetation, include the following. However, these topics have not proven amenable to WoS searches.

- Development or maintenance of native vegetation or areas of native flowers, especially within or near to areas where pollinator‐dependent crops are grown

Many recent articles, mostly associated with North America or Europe and rarely with Australasia, have pointed to how developing or maintaining areas of native vegetation or native plant species can develop or enhance reservoirs of pollinators, which can provide pollination services to adjacent or nearby crops. Recent such articles have included some that have arisen out of North America (e.g., Gervais et al., 2021; Grab et al., 2018; Olson et al., 2021) and some from Europe (e.g., Krimmer et al., 2019; Morrison et al., 2021; Pfister et al., 2018). Similar articles associated with Australasia have been relatively rare (e.g., Fijen et al., 2022; Howlett et al., 2021; New et al., 2021).

Similarly, some recent articles, mostly North American in origin, have described or illustrated how programs of ecological restoration that focus on plant communities may benefit bees and other pollinator species (e.g., Lane et al., 2020; Palmer, 2019; Tonietto & Larkin, 2018).

- Government restrictions or bans on the use of certain pesticides, such as neonicotinoids

The use of neonicotinoids, which are commonly used to reduce crop herbivory and have negative impacts on various non‐target animals including bees, has been recently banned or restricted in Europe and Canada (Demortain, 2021; Ogden, 2021; Vojvodic & Bazok, 2021). However, no such restrictions occur elsewhere, including in Australasia (Siviter, Johnson, et al., 2021).

- Integrated Pest and Pollinator Management

By focusing on actions that jointly benefit both control of crop pests and crop pollination and minimize conflicts between them, an Integrated Pest and Pollinator Management (IPPM) framework, applied in the context of agri‐ecosystems, may help to support both sustainable agricultual production and protection of pollinators. However, recent articles advocating this IPPM approach have arisen mostly in Europe (Belien et al., 2021; Egan et al., 2020; Lundin et al., 2021; Merle et al., 2022), North America (Bloom et al., 2022; Braman & Griffin, 2022; Leach et al., 2022; Okosun & Reddy, 2022; Pecenka et al., 2023; Penn et al., 2021), Africa (Adan et al., 2021; Toukem et al., 2022; Waithaka et al., 2023; Wangithi et al., 2022), and Asia (Jung, 2021; Jung & Shin, 2022; Wyckhuys et al., 2023), with none arising in Australasia.

- Sustainable agriculture and pollination

There has also been much discussion of pollination and pollinators in the context of sustainable agriculture in North America, Europe, and other parts of the world, but little in Australasia. For example, published articles discussing sustainable agriculture and arising in North America and Europe have often included consideration of pollinators or pollination (e.g., Batra, 1995; Kevan et al., 1990; Wentling et al., 2021) and occasionally in Africa, South America, and Asia (e.g., Garibaldi et al., 2011; Mkenda et al., 2019; Rehman et al., 2022), but rarely in Australasia (e.g., Triplett et al., 2012).

- First Nations knowledge and partnerships are plants, flowers, bees, and pollination and their conservation and management

First Nations people, throughout the world, have had connections, in some cases for thousands of years, with plants and bees, and may therefore possess knowledge relevant to pollination. For example, plants, including their flowers, have been sources of food and traditional medicine for First Nations people (Lorion & Small, 2021; Rice et al., 2011; Singha et al., 2021), including for Australian Aboriginal people (Gericke et al., 2021). And honey has been collected from the nests of social bees by Australian Aboriginal people (Fijn & Baynes‐Rock, 2018) and other First Nations people (Matias et al., 2019). First Nations people, including those in Australia and the rest of Australasia, may therefore possess useful and important information in relation to pollination, but we are not aware of any such studies. Similarly, partnerships involving Australian Aboriginal people and other First Nations people may benefit pollination‐related conservation and management.

Thus, yet again, as demonstrated by this brief review, attention to potential actions, that might mitigate the pollination crisis, has recently increased and mostly arisen in Europe and North America, with little such focus in Australia or NZ or the rest of Australasia.

This relative lack of activity and apparent interest in Australasia might thus suggest that the pollination crisis is not a problem in this region. After all, if no such problem exists, steps to address or mitigate it can hardly be warranted or expected!

However, this apparent difference between Australasia and other regions, especially Europe and North America, is relative and not absolute, and relevant research in relation to the pollination crisis has been carried out based in Australia, New Zealand, and Fiji. Recent examples include our own research (e.g., Prendergast, 2023; Prendergast & Ollerton, 2021; Prendergast, Tomlinson, et al., 2022) as well as the following: Australia (Cunningham et al., 2022; Geyle et al., 2021; Goodwin et al., 2021; Hogendoorn et al., 2021; Legge et al., 2023; New et al., 2021; Sanderson et al., 2021); New Zealand (Howlett et al., 2021; Stavert et al., 2018); and Fiji (Naaz et al., 2022).

### 3.6 Relevance in Australasia of factors underlying the pollination crisis

The above‐mentioned factors that are considered to underlie the pollination crisis are most likely extremely relevant to Australia, NZ, and the rest of Australasia. This is illustrated by the following examples.

For Australasia, both the area devoted to agriculture and the proportion of total land area used by agriculture are high compared with other countries and regions. The area used for agriculture in Australasia is similar to such areas for Europe, North America, and South America (Table 4). Similarly high proportions of total land area devoted to agriculture occur in Asia and Australasia (i.e., 51.7% and 46.3%, respectively; Table 4).

**TABLE 4:** Presented here, for each continental region, are land areas devoted to all agriculture and crops in particular, along with total land area, amounts of insecticide used and number of known bee species.

Region	Agricultural land (million ha)	Total land area (million ha)	% Agricultural land	Crop area (million ha)	Insecticide used (1000 kg)	Insecticide application rate (kg/ha)	# Bee species	% Worldwide species^a^
Europe	473.6	2401.1	19.7	287.9	70.5	0.24	3283	16.0
North America	590.8	2240.7	26.4	231.9	86.0	0.37	5172	25.2
Asia	1670.9	3230.9	51.7	534.3	406.5	0.76	5536	27.0
South America	538.1	1781.0	30.2	132.4	92.6	0.70	4069	19.8
Africa	1108.9	2987.4	37.1	203.8	27.7	0.14	3937	19.2
Australasia	396.3	855.0	46.3	32.9	14.9	0.45	1976	9.6
Oceania	0.3	1.1	23.4	0.09	0.006	0.07	69	0.3
Total/ Worldwide	4778.9	13,497.2	35.4	1423.3	698.2	0.49	20,507	

*Note*: Calculated from these values are the % total land area devoted to agriculture, application rate of insecticide in kg/ha, and % of known worldwide bee fauna. Australasia is considered to include Australia, Fiji, New Caledonia, New Zealand, Norfolk Island, New Guinea, Solomon Islands, and Vanuatu. Other islands of the South Pacific are included in Oceania.

^a^ %'s add to more than 100% because some bee species occur in more than one region.

Similarly, the rates of application of insecticide on crops per ha are relatively high in Australasia. This application rate in 2019 was 0.46 kg/ha in Australasia (see Table 4), which was higher than the rates in Europe and North America (i.e., 0.24 and 0.37 kg/ha, respectively; Table 4) and exceeded only by the rates in Asia and South America (i.e., 0.76 and 0.70 kg/ha, respectively; Table 4).

**FIGURE 1:**
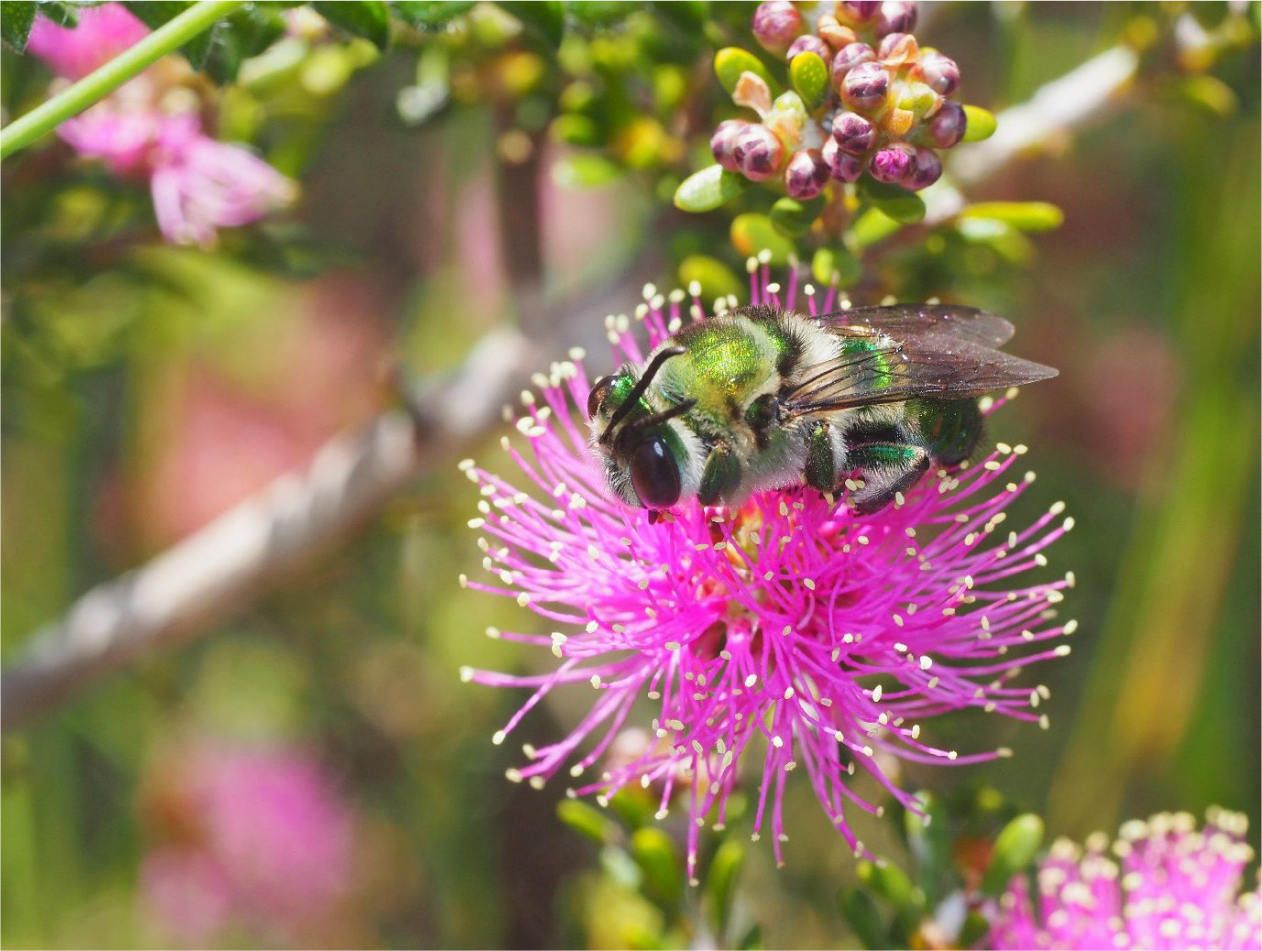
Australian bee (*Ctenocolletes smaragdinus*), from an endemic family (Stenotritidae), feeding on nectar from flower of *Melaleuca* sp (Myrtaceae) in southwest Western Australia, an internationally recognized biodiversity hotspot for bees and other organisms (Photo: Kit Prendergast).

Species diversity for bees is high in Australasia, with this region accounting for about 2000 known bee species, which is almost 10% of described species worldwide (i.e., 1990 known species in Australasia, out of 20,507 described species worldwide) (Table 4, based on https://www.discoverlife.org/mp/20q?guide=Apoidea_species&flags=HAS, accessed 26 June 2022; Orr et al., 2021). Importantly, the species in Australia are largely endemic (Figure 1), and many specialised plant–pollinator interactions occur (Houston, 2018). So, there is considerable opportunity for Australasian bee species to be adversely impacted as part of the pollination crisis.

Feral honeybees abound in Australasia, including in Australia, NZ, and Fiji, to the extent that, except in extremely arid areas, or relatively small areas that experience extreme winter cold, honeybees are encountered everywhere in large numbers, visiting flowers of native and introduced plant species and often outnumbering native bees and other flower visiting species (Howlett & Donovan, 2010; Naaz et al., 2022; Prendergast, Dixon, et al., 2022a). Indeed, it is difficult to find sites in Australia where the density of feral honeybees is low (Pyke & Balzer, 1985; Sugden & Pyke, 1991). So, in Australasia, there is also much potential for competition between feral honeybees and wild, native bees.

Furthermore, recorded densities of feral honeybees have been at least as high in Australasia as in other parts of the world, and so the potential for negative environmental impacts from feral honeybees is likely as high or higher in Australasia than elsewhere. For example, estimates of feral honeybee density depend on adopted methodology but, when the same methods are employed, density estimates for Australasia are relatively high compared with other areas (Cunningham et al., 2022; Oldroyd et al., 1997).

In other words, there is no reason to think that Australasia should be subject any less to the pollination crisis than is occurring elsewhere, and many reasons to think otherwise.

### 3.7 Does the pollination crisis warrant increased attention in Australasia?

There is therefore considerable cause for concern regarding the pollination crisis in Australasia and urgent need to take appropriate action to recognise, assess and mitigate the problem in Australasia. Because circumstances in Australasia are similar to those in Europe and North America, the lack of concern and evidence regarding the pollination crisis is likely the result of limited awareness rather than an absence of the problem.

Actions, arising from the above discussion, that could immediately help to address the pollination crisis in Australasia include the following:

- Taxonomic work to increase the numbers of described species for animals known or suspected to act as plant pollinators.
- Research aimed to determine nature and extent of changes that have occurred to the distributions and abundances of plant and animal species, with a focus on pollinators and pollination.
- Assessment of the conservation status of pollinator populations and legislative protection for those threatened with extinction.
- Establishment of long‐term monitoring of plant–pollinator relationships in diverse landscapes.
- Include pollinators and pollination in programs to achieve sustainable agriculture.
- Research aimed to determine the effects (including sublethal) of various pesticides (including insecticides such as neonicotinoids and herbicides such as glyphosate) on a range of pollinator species.
- Applying appropriate restrictions, including on the basis of evidence obtained elsewhere, to the use of various pesticides.
- Applying the Integrated Pest and Pollinator Management (IPPM) approach to agriculture.
- Increased partnerships with First Nations people in conservation and management of plants and their pollinators.

Of course, such actions could be encouraged and facilitated through Government regulation and funding.

### 3.8 Lack of appropriate policies and programs to deal with the pollination crisis

Appropriate policies and programs to deal with the pollination crisis are plentiful in Europe and North America, but absent from Australasia. In Europe, for example, a Common Agricultural Policy includes pollinator conservation (Cole et al., 2020; Hevia et al., 2021). Voluntary agreements through which landholders promote various aspects of nature conservation, including plant–pollinator relationships, are widespread across England (Image et al., 2022). In the USA, a considerable amount of legislation concerning various aspects of the pollination crisis has recently been enacted (Hall & Steiner, 2019), and there has even been a Presidential memorandum aimed at dealing with the issue (Althaus et al., 2021; Obama, 2014). Governments across Europe and North America have adopted strategies to conserve pollinators (Bloom et al., 2022; Schatz et al., 2021; Vasiliev, 2021). In addition, a number of studies have addressed the requirements for developing useful policy (Forister et al., 2019; Hill et al., 2019; Siviter et al., 2018; Sponsler et al., 2019). We are not aware of any similar policies or programs in Australasia.

However, while there have been no similar policies or programs for pollination‐related conservation in Australia, NZ, or elsewhere in Australasia, there have Down‐Under been some encouraging recent starts in related directions. A few special interest bee conservation groups have, for example, formed in Australia, though these mostly focus on honey bees (e.g., Wheen Bee Foundation – https://www.wheenbeefoundation.org.au/; Bee The Cure – Save The bees – https://www.beethecure.com.au/; Save Our Bees Australia – https://saveourbees.com.au/; Bee Day Australia – https://beedayaustralia.org.au/; all accessed July 2023). The Australian Native Bee Association (https://australiannativebee.org.au/; accessed July 2023), which was founded as recently as 2017, focuses on Australian native bees. Some of these groups are registered charities and encourage public donations; none is supported by government. Australian agencies, such as Agrifutures Australia and Hort Innovation, provide funding to support research and development for horticulture and other rural agriculture, including in relation to honeybees, crop cultivation, and pollination (e.g., https://agrifutures.com.au/knowledge‐hub/#stq=All&stp=1; https://www.horticulture.com.au/hort‐innovation/; both accessed July 2023). In New Zealand, a research program entitled “Operation Pollinator” has been jointly developed since 2019 by the Government owned New Zealand Institute for Plant and Food Research Ltd and the international agricultural company Syngenta to investigate relationships between pollinator diversity and crop yield for cultivated kiwi fruit (see https://www.plantandfood.com/en‐nz/article/operation‐pollinator‐tm‐in‐new‐zealand; accessed July 2023).

## 4 CONCLUSION

In other words, Australasia or Down‐Under is hardly likely to have “dodged the bullet” with regard to the pollination crisis, the time for appropriate policy and action is now, and there are many possible mitigating actions.

## AUTHOR CONTRIBUTIONS

**Graham H. Pyke:** Conceptualization (lead); investigation (lead); writing – original draft (lead); writing – review and editing (lead). **Kit S. Prendergast:** Conceptualization (supporting); investigation (supporting); writing – original draft (supporting); writing – review and editing (supporting). **Zong‐Xin Ren:** Conceptualization (supporting); investigation (supporting); writing – original draft (supporting); writing – review and editing (supporting).

## CONFLICT OF INTEREST STATEMENT

The authors declare no conflict of interest.

## Supporting information


Appendix S1

Appendix S2

## Data Availability

This article contains no original data.
