# Supplementary material for: Pollination crisis Down‐Under: Has Australasia dodged the bullet?
Source: Ecol Evol. 2023 Oct 30;13(11):e10639. doi: 10.1002/ece3.10639 (PMC10615657; doi:10.1002/ece3.10639)
Supplement: Supplementary file 1 — Appendix S1 [file ECE3-13-e10639-s001.docx]

Pollination crisis down-under – Has Australasia dodged the bullet?

Supplementary Material – Text

Issue S01: Concern regarding pollination crisis and pollinator crisis

Search engine: WoS – All Databases

Implemented by: GHP

Institution: Macquarie University

Date range: Up to end December 2021

Search target: Articles that refer to either ‘pollination crisis’ or ‘pollinator crisis’, and indicate associated concern

Search approach: Topic = “pollinat* crisis”

Search output details: See Supplementary Material – Spreadsheet – Worksheet #01 – WoS – Pollination Crisis

Search initial result: 91 references

# refs excluded from initial result: 4 refs (duplicates)

# refs analysed for publication year: 87

Years fully processed: All

# refs fully processed: 87

# refs excluded: 0

# refs analysed for country: 87

Results: Region

Table S01-1:

| Region | Frequency | % |
| --- | --- | --- |
| Europe | 36 | 41.4 |
| North America | 25 | 28.7 |
| Asia | 14 | 16.1 |
| South America | 9 | 10.3 |
| Africa | 2 | 2.3 |
| Australia | 0 | 0 |
| New Zealand | 1 | 1.1 |
| Rest of Australasia | 0 | 0 |
| TOTAL | 87 |  |

Results: Publication year

Table S01-2:

| Year Range | Frequency | N per yr |
| --- | --- | --- |
| 2020-2021 | 11 | 5.5 |
| 2015-2019 | 32 | 6.4 |
| 2010-2014 | 20 | 4.0 |
| 2005-2009 | 14 | 2.8 |
| 2000-2004 | 8 | 1.6 |
| 1995-1999 | 2 | 0.4 |
| 1990-1994 | 0 | 0 |
| 1980-1989 | 0 | 0 |
| 1970-1979 | 0 | 0 |
| TOTAL | 87 |  |

Issue S02: Concern regarding honeybee decline

Search engine: WoS – All Databases

Implemented by: GHP

Institution: Macquarie University

Date range: Up to end December 2021

Search target: Articles that refer to honeybee decline

Search approach: Topic = "honeybee* decline*" OR "honey bee* decline*" OR "decline* in honeybee*" OR "decline* in honey bee*" OR “decline* of honeybee*” OR “decline* of honey bee*”

Note: This search generated articles that related to honeybee decline, but a few also mentioned decline in other bee species

Search initial result: 122 references

# refs excluded from initial result: 2 (duplicates)

# refs analysed for publication year: 120

Years fully processed: 2011-2021

# refs fully processed: 102

# refs excluded: 0

# refs analysed for country/ region: 102

Search output details: See Supplementary Material – Spreadsheet – Worksheet #02 – WoS – Honeybee decline

Results: Region

Table S02-1:

| Region | Frequency | % |
| --- | --- | --- |
| Europe | 32 | 31.4 |
| North America | 40 | 39.2 |
| Asia | 18 | 17.6 |
| South America | 5 | 4.9 |
| Africa | 4 | 3.9 |
| Australia | 1 | 1.0 |
| New Zealand | 2 | 2.0 |
| Rest of Australasia | 0 | 0 |
| TOTAL | 102 |  |

Results: Publication year

Table S02-2:

| Year Range | Frequency | N per yr |
| --- | --- | --- |
| 2020-2021 | 20 | 10.0 |
| 2015-2019 | 57 | 10.4 |
| 2010-2014 | 34 | 6.8 |
| 2005-2009 | 7 | 1.4 |
| 2000-2004 | 0 | 0 |
| 1995-1999 | 1 | 0.2 |
| 1990-1994 | 1 | 0.2 |
| 1980-1989 | 0 | 0 |
| 1970-1979 | 0 | 0 |
| TOTAL | 120 |  |

Issue S03: Colony Collapse Disorder

Search engine: WoS – All Databases

Implemented by: GHP

Institution: Macquarie University

Date range: Up to end December 2021

Search target: Articles that express concern regarding ‘colony collapse disorder’ and refer to honeybees

Search approach: Topic = “colony collapse disorder” & Topic = “*Apis mellifera*”

Search initial result: 387 articles

# refs excluded from initial result: 4 duplicates

# refs analysed for publication year: 383

Years fully processed: 2017-2021

# refs fully processed: 122

# refs excluded: 4 (2 duplicates; 2 unavailable)

# refs analysed for country/ region: 118

Search output details: See Supplementary Material – Spreadsheet – Worksheet #03 – WoS – Colony Collapse Disorder (n=118 refs)

Results: Region

Table S03-1:

| Region | Frequency | % |
| --- | --- | --- |
| Europe | 36 | 30.5 |
| North America | 41 | 34.7 |
| Asia | 21 | 17.8 |
| South America | 14 | 11.9 |
| Africa | 3 | 2.5 |
| Australia | 3 | 2.5 |
| New Zealand | 0 | 0 |
| Rest of Australasia | 0 | 0 |
| TOTAL | 118 |  |

Results: Publication year

Table S03-2:

| Year Range | Frequency | N per yr |
| --- | --- | --- |
| 2020-2021 | 51 | 25.5 |
| 2015-2019 | 151 | 30.2 |
| 2010-2014 | 150 | 30.0 |
| 2005-2009 | 31 | 6.2 |
| 2000-2004 | 0 | 0 |
| 1995-1999 | 0 |  |
| 1990-1994 | 0 |  |
| 1980-1989 | 0 |  |
| 1970-1979 | 0 |  |
| TOTAL | 383 |  |

Issue S04: Concern regarding decline in bees other than honeybees

Search engine: WoS – All Databases

Implemented by: GHP

Institution: Macquarie University

Date range: Up to end December 2021

Search target: Articles that refer to bee decline but not honeybee decline

Search approach: Topic = "bee decline*" OR “bees decline*”

BUT NOT

Topic = "honeybee* decline*" OR "honey bee* decline*" OR "decline* in honeybee*" OR "decline* in honey bee*" OR “decline* of honeybee*” OR “decline* of honey bee*”

Note: Most of the articles generated by this search expressed concern about declines in bees other than honeybees. However, because some authors refer to honeybees simply as bees, some articles included or focused on honeybee decline. We did not attempt to exclude such articles.

Search initial result: 191 references

# refs excluded from initial result: 2 (duplicates)

# refs analysed for publication year: 189

Years fully processed: 2018-2021

# refs fully processed: 103

# refs excluded: 0

# refs analysed for country/ region: 103

Search output details: See Supplementary Material – Spreadsheet – Worksheet #04 – WoS – Bee decline excl honeybee decline

Results: Region

Table S04-1:

| Region | Frequency | % |
| --- | --- | --- |
| Europe | 35 | 34.0 |
| North America | 50 | 48.5 |
| Asia | 8 | 7.8 |
| South America | 5 | 4.9 |
| Africa | 1 | 1.0 |
| Australia | 4 | 3.9 |
| New Zealand | 0 | 0 |
| Rest of Australasia | 0 | 0 |
| TOTAL | 103 |  |

Results: Publication year

Table S04-2:

| Year Range | Frequency | N per yr |
| --- | --- | --- |
| 2020-2021 | 60 | 30.0 |
| 2015-2019 | 88 | 17.6 |
| 2010-2014 | 24 | 4.8 |
| 2005-2009 | 9 | 1.8 |
| 2000-2004 | 1 | 0.2 |
| 1995-1999 | 4 | 0.6 |
| 1990-1994 | 1 | 0.2 |
| 1980-1989 | 1 | 0.1 |
| 1970-1979 | 1 | 0.1 |
| TOTAL | 189 |  |

Issue S05: Numbers of threatened bee species in different geographic regions

Search engine: IUCN Red List (<https://www.iucnredlist.org>)

Implemented by: ZXR, with help from Yu Gao

Institution: Kunming Institute of Botany

Date accessed: 15 June 2022

Search target: Bee species considered threatened

Search approach: Each bee species considered threatened was assigned to one or more geographical regions

Results: Region

Table S05-1:

| Region | Frequency | % |
| --- | --- | --- |
| Europe | 402 | 83% |
| North America | 53 | 11% |
| Asia | 1 | 0% |
| Europe, Asia & North America | 5 | 1% |
| South America | 22 | 5% |
| Africa | 0 | 0 |
| Australia | 0 | 0 |
| New Zealand | 0 | 0 |
| Rest of Australasia | 0 | 0 |
| TOTAL | 483 |  |

Issue S06: Concern regarding food security in context of pollination

Search engine: WoS – All Databases

Implemented by: GHP

Institution: Macquarie University

Date range: Up to end December 2021

Search target: Articles that refer to ‘food security’ and ‘pollination’, and indicate concern regarding food security

Search approach: Topic = “food *security” AND Topic = pollination

Search initial result: 289 references

# refs excluded from initial result: 2 references (date extreme outlier; irrelevant)

# refs analysed for publication year: 287

Years fully processed: 2019-2022

# refs fully processed: 129

# refs excluded: 14 (12 considered irrelevant and 2 where neither relevance nor country could be determined)

# refs analysed for country/ region: 115

Search output details: See Supplementary Material – Spreadsheet – Worksheet #06 – WoS – Food security

Results: Region

Table S06-1:

| Region | Frequency | % |
| --- | --- | --- |
| Europe | 39 | 33.9 |
| North America | 20 | 17.4 |
| Asia | 22 | 19.1 |
| South America | 17 | 14.8 |
| Africa | 14 | 12.2 |
| Australia | 3 | 2.6 |
| New Zealand | 0 | 0 |
| Rest of Australasia | 0 | 0 |
| TOTAL | 115 |  |

Results: Publication year

Table S06-2:

| Year Range | Frequency | N per yr |
| --- | --- | --- |
| 2020-2021 | 97 | 48.5 |
| 2015-2019 | 125 | 25.0 |
| 2010-2014 | 58 | 11.6 |
| 2005-2009 | 6 | 1.2 |
| 2000-2004 | 1 | 0.2 |
| 1995-1999 | 0 | 0 |
| 1990-1994 | 0 |  |
| 1980-1989 | 0 |  |
| 1970-1979 | 0 |  |
| TOTAL | 287 |  |

Issue S07: Concern regarding pollination services decline

Search engine: WoS – All Databases

Implemented by: GHP

Institution: Macquarie University

Date range: Up to end December 2021

Search target: Articles that refer to decline in pollination services provided to plants by animals

Search approach: Topic = “pollination decline*” OR Topic = “pollination service* decline* OR Topic = “decline* in pollination service*” OR Topic = “pollination supply”, excluding articles relating to other aspects of pollination

Search initial result: 49 references

# refs excluded from initial result: 7 (6 irrelevant; 1 copy unavailable)

# refs analysed for publication year: 42

Years fully processed: All

# refs fully processed: 42

# refs excluded: 0

# refs analysed for country/ region: 42

Search output details: See Supplementary Material – Spreadsheet – Worksheet #07 – WoS – Decline in pollination services

Results: Region

Table S07-1:

| Region | Frequency | % |
| --- | --- | --- |
| Europe | 16 | 38% |
| North America | 12 | 29% |
| Asia | 6 | 14% |
| South America | 3 | 7% |
| Africa | 1 | 2% |
| Australia | 2 | 5% |
| New Zealand | 2 | 5% |
| Rest of Australasia | 0 | 0% |
| TOTAL | 42 |  |

Results: Publication year

Table S07-2:

| Year Range | Frequency | N per yr |
| --- | --- | --- |
| 2020-2021 | 11 | 5.5 |
| 2015-2019 | 15 | 3.0 |
| 2010-2014 | 12 | 2.4 |
| 2005-2009 | 1 | 0.2 |
| 2000-2004 | 3 | 0.6 |
| 1995-1999 | 0 | 0 |
| 1990-1994 | 0 |  |
| 1980-1989 | 0 |  |
| 1970-1979 | 0 |  |
| TOTAL | 42 |  |

Issue S08: Concern regarding pollination shortfall, gap or deficit in context of crop pollination

Search engine: WoS – All Databases

Implemented by: GHP

Institution: Macquarie University

Date range: Up to end December 2021

Search target: Articles that refer to pollination shortfall OR shortfall in pollination etc

Search approach: Topic = “pollination shortfall*” OR “pollination gap*” OR “pollination deficit*” OR “shortfall* in pollination” OR “gap* in pollination” OR “deficit* in pollination”

AND

Topic = crop

Search initial result: 84 references

# refs excluded from initial result: 1 (irrelevant)

# refs analysed for publication year: 83

Years fully processed: 1988 to 2021

# refs fully processed: 83

# refs excluded: 0

# refs analysed for country/ region: 83

Search output details: See Supplementary Material – Spreadsheet – Worksheet #08 – WoS – Pollination shortfall

Results: Region

Table S08-1:

| Region | Frequency | % |
| --- | --- | --- |
| Europe | 27 | 32.5 |
| North America | 15 | 18.1 |
| Asia | 13 | 15.7 |
| South America | 18 | 21.7 |
| Africa | 6 | 7.2 |
| Australia | 3 | 3.6 |
| New Zealand | 0 | 0 |
| Rest of Australasia | 1 (Fiji) | 1.2 |
| TOTAL | 83 |  |

Results: Publication year

Table S08-2:

| Year Range | Frequency | N per yr |
| --- | --- | --- |
| 2020-2021 | 21 | 10.5 |
| 2015-2019 | 30 | 6.0 |
| 2010-2014 | 27 | 5.4 |
| 2005-2009 | 2 | 0.4 |
| 2000-2004 | 1 | 0.2 |
| 1995-1999 | 0 | 0 |
| 1990-1994 | 1 | 0.2 |
| 1980-1989 | 1 | 0.1 |
| 1970-1979 | 0 | 0 |
| TOTAL | 83 |  |

Issue S09: Concern re Agricultural Intensification in context of Pollination

Search engine: WoS – All Databases

Implemented by: GHP

Institution: Macquarie University

Date range: Up to end December 2021

Search target: Articles that refer to Agricultural Intensification AND Pollination

Search approach: Topic = “agricultural intensification” AND Topic = “pollination”

Search initial result: 286

# refs excluded from initial result: 1 (duplicate)

# refs analysed for publication year: 285

Years fully processed: 2018-2021

# refs fully processed: 116

# refs excluded: 2 (1 duplicate; 1 irrelevant)

# refs analysed for country/ region: 114

Search output details: See Supplementary Material – Spreadsheet – Worksheet #09 – WoS – Agricultural Intensification

Results: Region

Table S09-1:

| Region | Frequency | % |
| --- | --- | --- |
| Europe | 60 | 53% |
| North America | 26 | 23% |
| Asia | 7 | 6% |
| South America | 12 | 11% |
| Africa | 8 | 7% |
| Australia | 0 | 0% |
| New Zealand | 1 | 1% |
| Rest of Australasia | 0 | 0% |
| TOTAL | 114 |  |

Results: Publication year

Table S09-2:

| Year Range | Frequency | N per yr |
| --- | --- | --- |
| 2020-2021 | 60 | 30.0 |
| 2015-2019 | 123 | 24.6 |
| 2010-2014 | 66 | 13.2 |
| 2005-2009 | 29 | 5.8 |
| 2000-2004 | 6 | 1.2 |
| 1995-1999 | 1 | 0.2 |
| 1990-1994 | 0 | 0 |
| 1980-1989 | 0 |  |
| TOTAL | 285 |  |

Issue S10: Evidence of sub-lethal impacts of neonics on non-Apis bees

Search engine: WoS – All Databases

Implemented by: Siviter et al 2021b, extended by GHP

Institution: Extension at Macquarie University

Date range: Up to part 2021, extended to end 2021

Search target: References providing evidence of impacts of neonics on non-Apis bees

Search approach: Topics employed by Siviter et al 2021 and current authors were as follows:

Topic = (“pesticide*” OR “insecticide*” OR “neonicotinoid*” OR "neonic*" OR “imidacloprid” OR “thiacloprid” OR “thiamethoxam” OR “clothianidin” OR “acetamiprid” OR “dinotefuran” OR “nitenpyram”)

AND

Topic = (“bumblebee*” OR “bumble bee*” OR “*Bombus*” OR “wild bee” OR “native bee” OR “*Osmia*” OR “*Megachile*” OR “wild bees” OR “solitary bee” OR “solitary bees” OR “*Scaptotrigona*” OR “stingless bee” OR “*Melipona*” OR “*Plebeia*” OR “mason” OR “squash bee” OR “squash bees” OR “*Peponapis*” OR “*Lasioglossum*” OR “sweat bee” OR “sweat bees” OR “*Frieseomelitta*” OR “*Tetragonisca*” OR “*Andrena*” OR “*Chelostoma*” OR “*Megachile rotundata” OR “Nomia melanderi” OR “Nomia” OR “Eucera pruinosa” OR “Eucera” OR “Nannotrigona*” OR “sting less bee*” OR “sting-less bee*” OR “*Trigona*” OR “*Megachilidae*” OR “*Andrenidae*” OR “*Halictidae*” OR “*Colletidae*” OR “*Stenotritidae*” OR “*Melittidae*” OR “*Anthophila*”)

AND

Topic = (“health” OR “sub-lethal” OR “sub lethal” OR “reproductive output” OR “reproduction” OR “cost” OR “fitness” OR “brood” OR “ovary development” OR “colony growth” OR “queen survival” OR “foraging” OR “performance” OR “mass” OR “mortality” OR “survival” OR “species richness” OR “monitoring” OR “assessment” OR *“*behavior” OR “behaviour” OR “foraging” OR “pollen” OR “toxicity” OR “pathogen”)

Search initial result: 604 references (Siviter et al 2021)

Exclusion criteria: Neonic concentration within observed ranges; presence of control treatments

# refs excluded from initial result: 551 (Siviter et al 2021)

# refs added by authors: 8

# refs analysed for publication year: 61

# refs analysed for country/ region: 61

Search output details: See Siviter et al 2021 (53 refs) and Supplementary Material – Spreadsheet – Worksheet #10 – WoS – Neonics sublethal (8 refs)

Results: Region

Table S10-1:

| Region | Frequency | % |
| --- | --- | --- |
| Europe | 40 | 65.6 |
| North America | 19 | 31.1 |
| Asia | 0 | 0 |
| South America | 2 | 3.3 |
| Africa | 0 | 0 |
| Australia | 0 | 0 |
| New Zealand | 0 | 0 |
| Rest of Australasia | 0 | 0 |
| TOTAL | 61 |  |

Results: Publication year

Table S10-2:

| Year Range | Frequency | N per yr |
| --- | --- | --- |
| 2020-2021 | 18 | 9.0 |
| 2015-2019 | 25 | 5.0 |
| 2010-2014 | 13 | 2.6 |
| 2005-2009 | 1 | 0.2 |
| 2000-2004 | 4 | 0.8 |
| 1995-1999 | 0 | 0 |
| 1990-1994 | 0 | 0 |
| 1980-1989 | 0 | 0 |
| TOTAL | 61 |  |

Issue S11: Concern regarding impact of urbanization on bee abundance & diversity

Search engine: Google Scholar

Implemented by: Prendergast et al 2022b

Institution: Curtin University, Western Australia

Date range: August 2016 to December 2019, but also two with 2020 publication date

Search target: Responses of wild bees to urbanisation

Search approach: Combinations of the terms: ‘bees, pollinators, insects, arthropods, native bees, wild bees’ combined with ‘urbanisation, cities, urban, land-use change, suburban, metropolis’ (Prendergast et al 2022)

Search output details: See Prendergast et al 2022. Supplementary Materials S1.

Search initial result: 215 studies from 202 references (incorrectly stated as 198 distinct references in Prendergast et al 2022)

# refs excluded from initial result: 13 duplicates

# refs analysed for publication year: 202

Years fully processed: To end 2019, plus part 2020

# refs fully processed: 202

# refs excluded: 0

# refs analysed for geographic region: 202

Adjustments re geographic region: For present analyses, we made the following adjustments: Costa Rica and Mexico are part of N. America, not S. America; Turkey and other Middle East countries are part of Asia, not Europe; Kemerovo, in eastern Russia, is in Asia, not Europe.

Results: Region

Table S11-1:

| Region | Frequency | % |
| --- | --- | --- |
| Europe | 75 | 37.1 |
| North America | 84 | 41.6 |
| Asia | 9 | 4.5 |
| South America | 20 | 9.9 |
| Africa | 2 | 1.0 |
| Australia | 12 | 5.9 |
| New Zealand | 0 | 0 |
| Rest of Australasia | 0 | 0 |
| TOTAL | 202 |  |

Results: Publication year

Table S11-2:

| Year Range | Frequency | N per yr |
| --- | --- | --- |
| Part 2020 | 2 |  |
| 2015-2019 | 105 | 21.0 |
| 2010-2014 | 56 | 11.2 |
| 2005-2009 | 28 | 5.6 |
| 2000-2004 | 8 | 1.6 |
| 1995-1999 | 1 | 0.2 |
| 1990-1994 | 2 | 0.4 |
| 1980-1989 | 0 | 0 |
| TOTAL | 202 |  |

Issue S12: Interest in Pollination Networks

Search engine: WoS – All Databases

Implemented by: GHP

Institution: Macquarie University

Date range: Up to end December 2021

Search target: Articles that refer to Pollination Networks

Search approach: Topic = “pollination network*”

Search initial result: 457

# refs excluded from initial result: 5 (duplicates)

# refs analysed for publication year: 452

Years fully processed: 2020-2021

# refs fully processed: 102

# refs excluded: 3 (2 duplicates; 1 irrelevant)

# refs analysed for country/ region: 99

Search output details: See Supplementary Material – Spreadsheet – Worksheet #12 – WoS – Pollination Networks

Results: Region

Table S12-1:

| Region | Frequency | % |
| --- | --- | --- |
| Europe | 33 | 33.3 |
| North America | 21 | 21.2 |
| Asia | 19 | 19.2 |
| South America | 15 | 15.2 |
| Africa | 5 | 5.1 |
| Australia | 2 | 2.0 |
| New Zealand | 2 | 2.0 |
| Rest of Australasia | 2 (Fiji 1; New Caledonia 1) | 2.0 |
| TOTAL | 99 |  |

Results: Publication year

Table S12-2:

| Year Range | Frequency | N per yr |
| --- | --- | --- |
| 2020-2021 | 100 | 50.0 |
| 2015-2019 | 207 | 41.4 |
| 2010-2014 | 104 | 20.8 |
| 2005-2009 | 37 | 7.4 |
| 2000-2004 | 3 | 0.6 |
| 1995-1999 | 0 | 0 |
| 1990-1994 | 1 | 0.2 |
| 1980-1989 | 0 | 0 |
| TOTAL | 452 |  |

Issue S13: Concern regarding taxonomic impediment in context of pollination

Search engine: WoS – All Databases

Implemented by: GHP

Institution: Macquarie University

Date range: Up to end December 2021

Search target: References expressing concern regarding taxonomic impediment, or equivalent, in context of pollination

Search approach: Topic=“pollinat*”

AND

Topic = "taxonom* impediment" OR "taxonom* knowledge" OR "taxonom* expertise" OR “taxonom* training” OR "taxonom* research" OR “unresolved taxonom*” OR “limited taxonom*”

Following topics were also tried but they yielded zero references:

"taxonom* gap" "taxonom* urgency" “taxonom* deficit” “taxonom* shortfall” “taxonom* problem” “taxonom* dilemma”

Search initial result: 84 references

# refs excluded from initial result: 60 (irrelevant)

# refs analysed for publication year: 24

Years fully processed: All

# refs fully processed: 24

# refs excluded: 0

# refs analysed for country/ region: 24

Search output details: See Supplementary Material – Spreadsheet – Worksheet #13 – WoS – taxonomic impediment

Results: Region

Table S13-1:

| Region | Frequency | % |
| --- | --- | --- |
| Europe | 3 | 12.5 |
| North America | 8 | 33.3 |
| Asia | 3 | 12.5 |
| South America | 5 | 20.8 |
| Africa | 2 | 8.3 |
| Australia | 2 | 8.3 |
| New Zealand | 1 | 4.2 |
| Rest of Australasia | 0 | 0 |
| TOTAL | 24 |  |

Results: Publication year

Table S13-2:

| Year Range | Frequency | N per yr |
| --- | --- | --- |
| 2020-2021 | 6 | 3.0 |
| 2015-2019 | 10 | 2.0 |
| 2010-2014 | 4 | 0.8 |
| 2005-2009 | 4 | 0.8 |
| 2000-2004 | 0 | 0 |
| 1995-1999 | 0 | 0 |
| 1990-1994 | 0 | 0 |
| 1980-1989 | 0 | 0 |
| TOTAL | 24 |  |
